# Notification of bacterial strains made available by the United Kingdom National Collection of Type Cultures in 2022

**DOI:** 10.1099/acmi.0.000756.v3

**Published:** 2024-07-05

**Authors:** Jake David Turnbull, Jo Dicks, Rachael Adkin, Alexander Dickinson, Dorota Kaushal, Mojisola Semowo, The NCTC 2022 Depositors Cohort, Hannah McGregor, Sarah Alexander

**Affiliations:** 1 The National Collection of Type Cultures, UK Health Security Agency, 61 Colindale Avenue, Colindale, London, NW9 5EQ, UK; 2 The NCTC 2022 Depositors Cohort consists of individuals who deposited strains into the NCTC and those instrumental in preparing the strains for submission to the NCTC. The NCTC 2022 Depositors Cohort are: Kathy Bernard (ex. National Microbiology Laboratory, Public Health Agency of Canada, Winnipeg, Manitoba, Canada), Marie Chattaway (Gastrointestinal Bacteria Reference Unit, UK Health Security Agency, Colindale, London, UK), Ka Lip Chew (Department of Laboratory Medicine, National University Hospital, Singapore, Singapore), Rachel Gilroy (ex. Microbes in the Food Chain Group, Quadram Institute, Norwich Research Park, Norwich, UK), Harriet Gooch (John Innes Centre, Norwich, UK), Thi Thu Hao Van (Royal Melbourne Institute of Technology, Bundoora Campus, Bundoora, Victoria, Australia), Jane Hawkey (Monash Central Clinical School, The Burnet Institute, Melbourne, Australia), Jay Hinton (Institute of Infection, Veterinary and Ecological Sciences, University of Liverpool, UK), Katie Hopkins (Antimicrobial Resistance & Mechanisms Service, Antimicrobial Resistance and Healthcare Associated Infections Unit, UK Health Security Agency, Colindale, London, UK), Claire Jenkins (Gastrointestinal Bacteria Reference Unit, Public Health England, Colindale, London, UK), Rob Mariman (Rijksinstituut voor Volksgezondheid en Milieu (RIVM), the National Institute for Public Health and the Environment, Bilthoven, The Netherlands), Despoina Mavridou (Department of Molecular Biosciences, The University of Texas at Austin, Austin, TX, USA), Mark Pallen (Quadram Institute, Norwich Research Park, Norwich, UK), Gavin Paterson (Royal (Dick) School of Veterinary Studies, University of Edinburgh, Edinburgh, Scotland, UK), Blanca Perez Sepulveda (Institute of Infection, Veterinary and Ecological Sciences, University of Liverpool, UK), Zeli Shen (Massachusetts Institute of Technology, Cambridge, Massachusetts, USA), Sho Shimada (Toho University, Faculty of Medicine, Omorinishi, Ota-ku, Tokyo), Sooyeon Song (Department of Animal Science, Jeonbuk National University, Backje-daero, Deokjin-gu, Jeonju-si, Jeollabuk-do, Republic of Korea), Dmitriy Volokhov (US Food and Drug Administration, Center for Biologics Evaluation and Research, Silver Spring, Maryland, USA), Thomas Wood (Pennsylvania State University, University Park, Pennsylvania, USA)

**Keywords:** bacteria, pathogens, novel taxa, culture collection, The National Collection of Type Cultures

## Abstract

Here, we report on the one hundred and twenty-five bacterial strains made available by the National Collection of Type Cultures in 2022 alongside a commentary on the strains, their provenance and significance.

## Data availability

The full details for each strain (citations for the full taxon descriptions, strain provenance, validated growth conditions, a selected bibliography for each strain where relevant, etc.) can be obtained from the NCTC online catalogue. Links to the catalogue entry for each strain can be found in Table 1, as can the links to the requisite genomic data wherever it is available.

## Introduction

The National Collection of Type Cultures (NCTC) is composed of approximately 6 000 bacterial strains, including 900 type strains (the strains on which the description of microbial taxa are based), which are predominantly of medical and veterinary relevance. In 2023, the NCTC is in its one-hundred-and-fourth year of continuous operation and is hosted by the UK Health Security Agency (UKHSA).

Last year the NCTC published a list of the forty-seven strains made available in 2021 [1]. In continuation of this annual announcement, here we list and detail the one hundred and twenty-five bacterial strains accessioned into, and subsequently made available from, the NCTC in 2022.

## Methods

Requests for accession of bacterial strains into the NCTC’s main collection (that is accessible to the scientific community) are submitted by members of the biosciences community to the staff of the NCTC alongside a completed deposit form. Following review of the deposit form(s) and the details of the request, a decision is made as to whether it would be appropriate for the bacterial strain in question to be accessioned into the NCTC. Bacterial strains are only accepted into the NCTC following a review by the curatorial staff, e.g. that the strain is type strain or is within remit of the collection in being of medical and veterinary relevance.

Additionally, proactive requests are made by NCTC staff to collaborating culture collections and scientists worldwide for the accession of bacterial strains of interest to the NCTC and the wider scientific community at large. Where positive and affirmative responses are obtained for such requests, deposit forms are obtained and submitted for review by the curatorial staff of the NCTC as above.

Following communication of a formal acceptance of the bacterial strain into the NCTC to the depositing party, arrangements are made for shipment of the bacterial strain to the NCTC. Upon receipt, the bacterial strains are preserved, predominantly by freeze drying. The preserved strains are checked for viability, purity, and conformation to expected species level identification and select phenotypic properties using a variety of techniques. Some strains are further examined using bespoke genomic analysis, e.g. multi-locus sequence typing (MLST) and antimicrobial resistance gene (AMR) profiling. Where third party genomic data (typically accession identifiers from the major online nucleotide sequence archives such as GenBank and the European Nucleotide Archive) are available from the depositor or prior publications associated with the deposited strain, this information is recorded by the NCTC.

Following successful checks the strains are integrated into the NCTC online catalogues and inventory systems and are made available to the scientific community via the NCTC online catalogue. More information on depositing bacterial strains into the NCTC, a free service, is available on the NCTC / UKHSA Culture Collections website: https://www.culturecollections.org.uk.

## Results: bacterial strains made available by the NCTC in 2022

One hundred and twenty-five bacterial strains submitted from twenty-four different individuals across twenty institutes, predominantly from within the UK, were made available to the scientific community by the NCTC in 2022. All strains are listed in Table 1.

**Table 1.** The 125 strains made available by the NCTC in 2022, including 18 type strains of novel taxa, seven previously described type strains (including one AMR strain; NCTC 14468), 29 strains proposed as type strains for effectively described taxa, 12 AMR strains (including one previously described type strain; NCTC 14468), nine strains that carry linear plasmids (an atypical trait), two laboratory reference strains, 27 strains obtained from legacy archive and historical collections including 15 known to be from the pre-antibiotic era, 15 strains of human clinical significance and seven of veterinary provenance. The 25 type strains are denoted by ^T^ in the table above and throughout this manuscript. All strains are listed alongside their NCTC identifier, species name, primary reference, attribute, and whole genome sequence data where it is available through accessible databases. Whole genome sequence data listed above originate with the manuscripts in which they are described and are whole genome sequence assemblies where they are available. Where whole genome sequence assemblies are not available, shotgun contig collections, ENA read set archives, and NCBI BioProjects are listed, denoted by ^a, b^ and ^c^ respectively. For the nine strains that carry linear plasmids, the sequence data for the linear plasmid is given, as denoted by ^d^

NCTC ID.	Species name	Primary reference	Strain attribute	Available whole genome sequence data
NCTC 13866 ^T^	*Helicobacter enhydrae*	10	Type strain of novel taxon	CP016503.1
NCTC 14187 ^T^	*Campylobacter taeniopygiae*	11	Type strain of novel taxon	NXLY00000000.1^a^
NCTC 14266 ^T^	*Campylobacter estrildidarum*	11	Type strain of novel taxon	NXLZ00000000.1^a^
NCTC 14267 ^T^	*Campylobacter aviculae*	11	Type strain of novel taxon	NXMA00000000.1^a^
NCTC 14274 ^T^	*Enterobacter quasihormaechei*	3	Type strain of novel taxon	–
NCTC 14307 ^T^	*Mycoplasma enhydrae*	8	Type strain of novel taxon	–
NCTC 14344 ^T^	*Mycoplasma phocoenae*	9	Type strain of novel taxon	NZ_CP051481.1
NCTC 14419 ^T^	*Staphylococcus singaporensis*	5	Type strain of novel taxon	JABWHB000000000.1^a^
NCTC 14429 ^T^	*Mycoplasmsa miroungigenitalium*	9	Type strain of novel taxon	CP053096.1
NCTC 14430 ^T^	*Mycoplasma miroungirhinis*	9	Type strain of novel taxon	CP053097.1
NCTC 14448 ^T^	*Corynebacterium hindlerae*	6	Type strain of novel taxon	CP059833.1
NCTC 14449 ^T^	*Franklinella schreckenbergeri*	7	Type strain of novel taxon	GCA_003703475.1
NCTC 14450 ^T^	*Vandammella animalimorsus*	7	Type strain of novel taxon	GCA_002285285.1
NCTC 14452 ^T^	*Staphylococcus caledonicus*	15	Type strain of novel taxon	GCA_016238465.1
NCTC 14580 ^T^	*Pseudomonas tohonis*	4	Type strain of novel taxon	AP023189.1
NCTC 14581 ^T^	*Legionella antarctica*	2	Type strain of novel taxon	AP022839.1
NCTC 14608 ^T^	*Enterococcus innesii*	12	Type strain of novel taxon	GCA_018982785.1
NCTC 14611 ^T^	*Campylobacter bilis*	13	Type strain of novel taxon	WUED00000000.1^a^
NCTC 14545	‘Acinetobacter pecorum’	17	Effectively described taxon	GCA_014837015.1
NCTC 14546	‘Bacillus norwichensis’	17	Effectively described taxon	GCA_014836955.1
NCTC 14547	‘Brevibacterium gallinarum’	17	Effectively described taxon	GCA_014836885.1
NCTC 14549	‘Oerskovia douganii’	17	Effectively described taxon	GCA_015142735.1
NCTC 14550	‘Oerskovia gallyi’	17	Effectively described taxon	GCA_014836745.1
NCTC 14551	‘Sporosarcina gallistercoris’	17	Effectively described taxon	GCA_014836415.1
NCTC 14552	‘Planococcus wigleyi’	17	Effectively described taxon	GCA_014836985.1
NCTC 14553	‘Psychrobacter communis’	17	Effectively described taxon	GCA_014836505.1
NCTC 14554	‘Oerskovia merdavium’	17	Effectively described taxon	GCA_014836755.1
NCTC 14555	‘Ochrobacterum gallinarum’	17	Effectively described taxon	GCA_014836735.1
NCTC 14557	‘Oceanitalea stevensii’	17	Effectively described taxon	GCA_014837105.1
NCTC 14558	‘Brevundimonas guildfordensis’	17	Effectively described taxon	GCA_014836405.1
NCTC 14559	‘Arthrobacter pullicola’	17	Effectively described taxon	GCA_014836875.1
NCTC 14560	‘Arthrobacter gallicola’	17	Effectively described taxon	GCA_014836775.1
NCTC 14563	‘Kaistella pullorum’	17	Effectively described taxon	GCA_014837035.1
NCTC 14564	‘Cellulomonas avistercoris’	17	Effectively described taxon	GCA_014836445.1
NCTC 14567	‘Escherichia whittamii’	17	Effectively described taxon	GCA_014836715.1
NCTC 14569	‘Corynebacterium gallinarum’	17	Effectively described taxon	GCA_014837045.1
NCTC 14570	‘Luteimonas colneyensis’	17	Effectively described taxon	GCA_014836665.1
NCTC 14571	‘Comamonas avium’	17	Effectively described taxon	GCA_014836675.1
NCTC 14573	‘Paenibacillus gallinarum’	17	Effectively described taxon	GCA_014836635.1
NCTC 14576	‘Ureibacillus galli’	17	Effectively described taxon	GCA_014836845.1
NCTC 14577	‘Stenotrophomonas pennii’	17	Effectively described taxon	GCA_014836545.1
NCTC 14578	‘Solibacillus faecavium’	17	Effectively described taxon	GCA_014836905.1
NCTC 14618	‘Serpens gallinarum’	17	Effectively described taxon	GCA_014836765.1
NCTC 14237	‘Salmonella enterica subsp. hibernicus’	16	Effectively described taxon	–
NCTC 14238	‘Salmonella enterica subsp. hibernicus’	16	Effectively described taxon	–
NCTC 14239	‘Salmonella enterica subsp. essexiensis’	16	Effectively described taxon	–
NCTC 14240	‘Salmonella enterica subsp. essexiensis’	16	Effectively described taxon	–
NCTC 14631 ^T^	*Legionella norrlandica*	23	Type strain, previously validly published	JNCF01000000.1^a^
NCTC 14634 ^T^	*Streptococcus hyovaginalis*	19	Type strain, previously validly published	–
NCTC 14635 ^T^	*Streptococcus minor*	20	Type strain, previously validly published	–
NCTC 14682 ^T^	*Deinococcus radiodurans*	24	Type strain, previously validly published	–
NCTC 14694 ^T^	*Streptococcus pseudoporcinus*	21	Type strain, previously validly published	–
NCTC 14695 ^T^	*Streptococcus lactarius*	22	Type strain, previously validly published	–
NCTC 14468 ^T^	*Acinetobacter colistiniresistens*	26	Type strain, previously validly published / AMR	ATGK00000000.1^a^
NCTC 14380	*Escherichia coli*	28	Antimicrobial Resistant	–
NCTC 14476	*Escherichia coli*	–	Antimicrobial Resistant	–
NCTC 14477	*Escherichia coli*	–	Antimicrobial Resistant	–
NCTC 14579	*Staphylococcus aureus*	27	Antimicrobial Resistant	–
NCTC 14617	*Staphylococcus aureus*	–	Antimicrobial Resistant	–
NCTC 14622	*Salmonella enterica* subsp. *enterica* serotype Blockley	–	Antimicrobial Resistant	–
NCTC 14636	*Staphylococcus aureus*	–	Antimicrobial Resistant	–
NCTC 14637	*Staphylococcus aureus*	–	Antimicrobial Resistant	–
NCTC 14638	*Enterococcus faecium*	–	Antimicrobial Resistant	–
NCTC 14639	*Enterococcus faecalis*	–	Antimicrobial Resistant	–
NCTC 14663	*Proteus mirabilis*	–	Antimicrobial Resistant	–
NCTC 14478	*Pseudomonas aeruginosa*	30	Laboratory Reference Strain	AE004091.2
NCTC 14367	*Escherichia coli*	29	Laboratory Reference Strain	–
NCTC 14642	*Klebsiella pneumoniae*	31	Exhibits atypicality of interest	LR890180.1^d^
NCTC 14644	*Klebsiella pneumoniae*	31	Exhibits atypicality of interest	CP083770.1^d^
NCTC 14645	*Klebsiella variicola*	31	Exhibits atypicality of interest	LR890701.1^d^
NCTC 14646	*Klebsiella variicola*	31	Exhibits atypicality of interest	LR890402.1^d^
NCTC 14647	*Klebsiella variicola*	31	Exhibits atypicality of interest	CP083793.1^d^
NCTC 14648	*Klebsiella pneumoniae*	31	Exhibits atypicality of interest	CP083785.1^d^
NCTC 14649	*Klebsiella pneumoniae*	31	Exhibits atypicality of interest	CP083778.1^d^
NCTC 14650	*Klebsiella pneumoniae*	31	Exhibits atypicality of interest	LR890730.1^d^
NCTC 14651	*Klebsiella pneumoniae*	31	Exhibits atypicality of interest	LR890645.1^d^
NCTC 14665	*Bordetella pertussis*	38	Contemporary or significant clinical isolate	CP009751.1
NCTC 14294	*Yersinia enterocolitica*	39	Contemporary or significant clinical isolate	ABKTWF000000000.1^a^
NCTC 14401	*Mycoplasma hominis*	–	Contemporary or significant clinical isolate	–
NCTC 14420	*Staphylococcus singaporensis*	5	Contemporary or significant clinical isolate	JABWPO000000000.1^a^
NCTC 14421	*Staphylococcus singaporensis*	5	Contemporary or significant clinical isolate	JABWHF000000000.1^a^
NCTC 14422	*Staphylococcus singaporensis*	5	Contemporary or significant clinical isolate	JABWHE000000000.1^a^
NCTC 14423	*Staphylococcus singaporensis*	5	Contemporary or significant clinical isolate	JABWHD000000000.1^a^
NCTC 14424	*Staphylococcus singaporensis*	5	Contemporary or significant clinical isolate	JABWHC000000000.1^a^
NCTC 14672	*Salmonella enterica* subsp. *enterica* serotype Typhimurium	32	Contemporary or significant clinical isolate	GCF_000188735.1
NCTC 14673	*Salmonella enterica* subsp. *enterica* serotype Typhimurium	33	Contemporary or significant clinical isolate	ERA000075^b^
NCTC 14674	*Salmonella enterica* subsp. *enterica* serotype Enteritidis	34	Contemporary or significant clinical isolate	GCF_015241125.1
NCTC 14675	*Salmonella enterica* subsp. *enterica* serotype Enteritidis	35	Contemporary or significant clinical isolate	GCF_015241005.1
NCTC 14676	*Salmonella enterica* subsp. *enterica* serotype Enteritidis	34	Contemporary or significant clinical isolate	GCA_015240855.1
NCTC 14677	*Salmonella enterica* subsp. *enterica* serotype Typhimurium	33	Contemporary or significant clinical isolate	PRJEB28511^c^
NCTC 14678	*Salmonella enterica* subsp. *enterica* serotype Typhimurium	36	Contemporary or significant clinical isolate	ERR023634^b^
NCTC 14347	*Mycoplasma enhydrae*	8	Contemporary or significant veterinary isolate	NZ_JAFIRD000000000.1^a^
NCTC 14348	*Mycoplasma enhydrae*	8	Contemporary or significant veterinary isolate	–
NCTC 14349	*Mycoplasma enhydrae*	8	Contemporary or significant veterinary isolate	NZ_JAOXHI000000000.1^a^
NCTC 14350	*Mycoplasma enhydrae*	8	Contemporary or significant veterinary isolate	NZ_JAOXHH000000000.1^a^
NCTC 14425	*Mycoplasma anatis*	–	Contemporary or significant veterinary isolate	–
NCTC 14426	*Mycoplasma anatis*	–	Contemporary or significant veterinary isolate	–
NCTC 14439	*Mycoplasma miroungigenitalium*	9	Contemporary or significant veterinary isolate	NZ_JAHMHL000000000.1^a^
NCTC 14065	*Vibrio cholerae*	–	Obtained from a legacy strain archive	–
NCTC 14066	*Vibrio cholerae*	–	Obtained from a legacy strain archive	–
NCTC 14067	*Vibrio cholerae*	–	Obtained from a legacy strain archive	–
NCTC 14068	*Vibrio cholerae*	–	Obtained from a legacy strain archive	–
NCTC 14071	*Vibrio cholerae*	–	Obtained from a legacy strain archive	–
NCTC 14072	*Vibrio cholerae*	–	Obtained from a legacy strain archive	–
NCTC 14074	*Vibrio cholerae*	–	Obtained from a legacy strain archive	–
NCTC 14080	*Vibrio cholerae*	–	Obtained from a legacy strain archive	–
NCTC 14081	*Vibrio cholerae*	–	Obtained from a legacy strain archive	–
NCTC 14082	*Vibrio cholerae*	–	Obtained from a legacy strain archive	–
NCTC 14084	*Vibrio cholerae*	–	Obtained from a legacy strain archive	–
NCTC 14085	*Vibrio cholerae*	–	Obtained from a legacy strain archive	–
NCTC 14117	*Klebsiella pneumoniae*	25	Obtained from a legacy strain archive, Pre-Antibiotic Era Isolate	–
NCTC 14249	*Salmonella enterica* subsp. *enterica* serotype Poona	25	Obtained from a legacy strain archive, Pre-Antibiotic Era Isolate	ERS222747^b^
NCTC 14251	*Salmonella enterica* subsp. *enterica* serotype Heidelberg	25	Obtained from a legacy strain archive, Pre-Antibiotic Era Isolate	ERS203038^b^
NCTC 14252	*Salmonella enterica* subsp. *enterica* serotype Rostock	25	Obtained from a legacy strain archive, Pre-Antibiotic Era Isolate	ERS203052^b^
NCTC 14253	*Salmonella enterica* subsp. *enterica* serotype Enteritidis	25	Obtained from a legacy strain archive, Pre-Antibiotic Era Isolate	ERS203053^b^
NCTC 14256	*Salmonella enterica* subsp. *enterica* serotype Bareilly	25	Obtained from a legacy strain archive, Pre-Antibiotic Era Isolate	–
NCTC 14257	*Salmonella enterica* subsp. *enterica* serotype Newport	25	Obtained from a legacy strain archive, Pre-Antibiotic Era Isolate	ERS203087^b^
NCTC 14258	*Salmonella enterica* subsp. *enterica* serotype Gallinarum *var*. Gallinarum	25	Obtained from a legacy strain archive, Pre-Antibiotic Era Isolate	ERS203088^b^
NCTC 14259	*Salmonella enterica* subsp. *enterica* serotype Eastbourne	25	Obtained from a legacy strain archive, Pre-Antibiotic Era Isolate	–
NCTC 14260	*Salmonella enterica* subsp. *enterica* serotype Eastbourne	25	Obtained from a legacy strain archive, Pre-Antibiotic Era Isolate	ERS203093^b^
NCTC 14261	*Salmonella enterica* subsp. *enterica* serotype Anatum	25	Obtained from a legacy strain archive, Pre-Antibiotic Era Isolate	ERS203132^b^
NCTC 14262	*Salmonella enterica* subsp. *enterica* serotype Gallinarum *var*. Gallinarum	25	Obtained from a legacy strain archive, Pre-Antibiotic Era Isolate	ERS222816^b^
NCTC 14263	*Salmonella enterica* subsp. *enterica* serotype Muenchen	25	Obtained from a legacy strain archive, Pre-Antibiotic Era Isolate	ERS203157^b^
NCTC 14264	*Salmonella enterica* subsp. *enterica* serotype Muenchen	25	Obtained from a legacy strain archive, Pre-Antibiotic Era Isolate	ERS179685^b^
NCTC 14265	*Salmonella enterica* subsp. *salamae*	25	Obtained from a legacy strain archive, Pre-Antibiotic Era Isolate	ERS179701^b^

Of these one hundred and twenty-five strains, eighteen represent recently published novel prokaryotic taxa, seven are type strains of taxa previously validly published, and twenty-nine are proposed type strains, i.e. effectively described prokaryotic taxa; proposed novel taxa with descriptions published outside of the International Journal of Systematic and Evolutionary Microbiology. It is common for effectively described species to become validly described on inclusion in the frequently published IJSEM Validation Lists.

The remaining strains pertain to antimicrobial resistant strains (AMR; *n*=12), strains that exhibit atypical biology (in this case, they all carry linear plasmids; *n*=9), laboratory reference strains (*n*=2), those obtained from historic and legacy strain archives including known pre-antibiotic era isolates (*n*=27) and strains of clinical significance and veterinary provenance (*n*=22).

The full details for each strain (citations for the full taxon descriptions, strain provenance, validated growth conditions, typing information, etc.) can be obtained from the NCTC online catalogue.

## Discussion: commentary on the accessioned strains

### Validly described novel taxa and effectively described taxa

The novel taxa listed in Table 1 were all isolated from humans and animals (which were either healthy or diseased, with some instances of this information not being disclosed), with the exception of NCTC 14581^T^ *Legionella antarctica*, a strain which was isolated from lake sediment from the Naga-Ike Lake in Antarctica. NCTC 14581^T^ constitutes the first validly described psychrotolerant member of the genus *Legionella* [2].

NCTC 14274^T^ *Enterobacter quasihormaechei* [3] and NCTC 14580^T^ *Pseudomonas tohonis* [4] were isolated from human sputum and skin of a human burn wound patient respectively and are described to be opportunistically pathogenic. NCTC 14419^T^ *Staphylococcus singaporensis* was isolated from a human cholecystostomy specimen and determined to be a member of the *Staphylococcus aureus* complex within the species description [5]. Clinical features of infection (e.g. manifested soft tissue infection) were reported to be similar to that of *Staphylococcus aureus* despite a paucity of virulence genes typically associated with *Staphylococcus aureus*, specifically enterotoxin and Panton-Valentine leucocidin genes.

NCTC 14448^T^ *Corynebacterium hindlerae* [6], NCTC 14449^T^ *Franklinella schreckenbergeri* [7] and NCTC 14450^T^ *Vandammella animalimorsus* [7] were all deposited by the National Microbiology Laboratory of the Public Health Agency of Canada and were also isolated from substrates of human origin; a granuloma, cellulitis, and a peritoneal dialysate, respectively.

*Corynebacterium hindlerae* does not carry the diphtheria toxin (*tox*) gene or produce diphtheria toxin, however, the species bears strong morphological similarity to toxigenic *Corynebacterium* strains when grown on modified Tinsdale medium. The species descriptions for *Vandammella animalimorsus* and *Franklinella schreckenbergeri* do not hypothesize on the pathogenicity of either species, however strains of each species were reported to be detected in the oral microbiome of dogs and cats, with *Vandammella animalimorsus* having previously been isolated from wounds with links to animal bites. Both species descriptions constitute the first members of the novel genera *Franklinella* and *Vandammella* [7].

NCTC 14307^T^ *Mycoplasma enhydrae* [8] and NCTC 14344^T^ *Mycoplasma phocoenae* [9] were isolated from a Southern Sea Otter (*Enhydra lutris nereis*) and a Harbour Porpoise (*Phocoena phocoena*) respectively, whereas NCTC 14429^T^ *Mycoplasma miroungigenitalium* [9] and NCTC 14430^T^ *Mycoplasma miroungirhinis* [9] were both isolated from Northern Elephant Seals (*Mirounga angustirostris*). While comparative pathogenomic analysis identified no putative virulence factors within these four species, the authors who described them highlighted that the impact of many *Mycoplasma* species on their hosts is poorly understood and as such warrants further examination [9]. NCTC 13866^T^ *Helicobacter enhydrae* is of similar provenance of NCTC 14307^T^, having also been isolated from a Southern Sea Otter [10].

NCTC 14187^T^ *Campylobacter taeniopygiae*, NCTC 14266^T^ *Campylobacter estrildidarum* and NCTC 14267^T^ *Campylobacter aviculae* [11] were all isolated from the faeces of laboratory maintained Zebra Finch (*Taeniopygia guttata*) housed at the Massachusetts Institute of Technology that were presumed to have been infected by *Campylobacter jejuni*. While the birds themselves were not afflicted with any observed pathology, each of these new taxa are hypothesised to have zoonotic potential and may be pathogenic to humans as suggested by the presence of known virulence gene homologs within each microbial species. Similarly, NCTC 14608^T^ *Enterococcus innesii*, was also isolated from an animal that sees use in laboratory settings, commonly as a model organism in pathogenicity assays: the Greater Wax Moth (*Galleria mellonella*) [12].

NCTC 14611^T^ *Campylobacter bilis* [13] was isolated from the bile of a layer chicken with Spotty Liver Disease, an emerging disease in poultry, and the second novel *Campylobacter* species associated with the disease, the first being *Campylobacter hepaticus* [14], the type strain of which is held by the NCTC under accession identifier NCTC 13823^T^. NCTC 14452^T^ *Staphylococcus caledonicus* was isolated within a study into the commensal microbiota of healthy domestic dogs [15].

The valid descriptions of the above taxa were published in 2019 (*n*=2), 2020 (*n*=2), 2021 (*n*=9) and 2022 (*n*=5). In addition, twenty-nine strains were assigned to twenty-seven effectively described taxa.

Following a re-evaluation and proposed refinement of the taxonomic structure of the genus Salmonella NCTC 14237 and NCTC 14238 were proposed as type strains of *Salmonella enterica* subspecies IX (subsp. ‘hibernicus’) and NCTC 14239 and NCTC 14240 of subspecies X (subsp. ‘essexiensis’), and thereby accessioned into the NCTC [16].

The remaining twenty-five strains are proposed type strains, one for each of twenty-five different effectively described species all isolated from chicken faeces, the products of a single metagenomically informed study of the chicken gut microbiome [17]. Description of these proposed taxa help to elucidate a microbial ecology associated with an organism whose global population has an estimated combined biomass that is greater than all other bird species that currently exist in the Earth’s biosphere [18].

## Existing taxa

The previously described and validly published type strains of seven species were made available from the NCTC in 2022, facilitated by strain exchange with three European culture collections: the Czech National Collection of Type Cultures (CNCTC), the Culture Collection of the University of Gothenburg (CCUG) and the Deutsche Sammlung von Mikroorganismen und Zellkulturen (DSMZ). The accession of NCTC 14634^T^ *Streptococcus hyovaginalis* [19], NCTC 14635^T^ *Streptococcus minor* [20], NCTC 14694^T^ *Streptococcus pseudoporcinus* [21] and NCTC 14695^T^ *Streptococcus lactarius* [22] expand the number of *Streptococcus* species available from the NCTC that are relevant to human and animal health, as does the accession of NCTC 14631^T^ *Legionella norrlandica* [23] with regard to the genus *Legionella*.

NCTC 14682^T^ *Deinococcus radiodurans* [24], as its name indicates, is the type strain of a species which is among the most radiation resistant extant organisms; another radiation-resistant species being *Deinococcus radiophilus*, the type strain of which is available from the NCTC as NCTC 10785^T^. The species description for *Deinococcus radiodurans* was published by R. G. E. Murray in 1981, who also facilitated the transfer of his father’s pre-antibiotic era collection of *Enterobacteriaceae* [25] to the NCTC in the 1980s, which is discussed further below.

NCTC 14468^T^ *Acinetobacter colistiniresistens* is the type strain of a species intrinsically resistant to polymyxin antibiotics [26], as were all strains of the same species isolated within the study containing the taxon description, were from ‘clinical context free of colistin-based therapy’. NCTC 14468^T^ is confirmed as being phenotypically resistant to colistin with an MIC of >8 mg/L, as determined by the UKHSA Antimicrobial Resistance and Healthcare Associated Infections (AMRHAI) reference unit during the accession of this strain into the NCTC.

Five additional AMR strains were deposited by the AMRHAI laboratory, four of which (NCTC 14636 *Staphylococcus aureus*, NCTC 14637 *Staphylococcus aureus*, NCTC 14638 *Enterococcus faecium*, NCTC 14639 *Enterococcus faecalis*) are all resistant to the synthetic antibiotic linezolid as conferred by mutations within one or all copies of the 23S rRNA genes carried by each strain. NCTC 14663 *Proteus mirabilis*, also deposited by AMRHAI, was isolated from an instance of a UTI and is positive for the Ambler class D beta-lactamase OXA-23, however, is largely susceptible to carbapenem antibiotics. Such strains have previously been detected in Belgium and France, and where a lineage of OXA-23 positive *Proteus mirabilis* is hypothesized to have been circulating since 1996 [27].

NCTC 14579 *Staphylococcus aureus* [28] and NCTC 14380 *Escherichia coli* [29] are a part of two distinct sets of strains which were made available in 2021. NCTC 14579 is positive for the *mecC* gene, responsible for methicillin resistance, and is a member of a strain set along with NCTC 14457 to NCTC 14462 consecutively, NCTC 14464 and NCTC 14465, all *mecC* positive *Staphylococcus aureus* of human provenance. NCTC 14380 *Escherichia coli* is positive for the *mcr*-5 colistin resistance gene, and collates with NCTC 14376 to NCTC 14379 consecutively and NCTC 14381, which likewise all carry functional variants of *mcr*-1. NCTC 14579 and NCTC 14380 were described as AMR strains in last year’s notification of bacterial strains made available from the NCTC [1], where the provenance and context of these strains are detailed in more depth.

NCTC 14617 *Staphylococcus aureus* is a penicillin resistant strain, and NCTC 14622 *Salmonella enterica* subsp. *enterica* Serotype Blockley is resistant to fluoroquinolone class antibiotics and tetracycline. NCTC 14476 *Escherichia coli* and NCTC 14477 *Escherichia coli* both produce extended spectrum beta-lactamases, a plasmid-mediated AmpC and CTX-M-1 respectively.

NCTC 14367 *Escherichia coli* and NCTC 14478 *Pseudomonas aeruginosa* are both laboratory reference strains. NCTC 14367 is a part of a set of eleven *Escherichia coli* strains (NCTC 14364 to NCTC 14374 consecutively) that have had cryptic prophage removed and demonstrated that each prophage contributes to survival of the bacterial cell that carries it [30]. NCTC 14478 is the PAO1 strain of *Pseudomonas aeruginosa*, a moderately pathogenic isolate of the species. In the year 2000 the publication of the whole genome sequence of PAO1 was the largest bacterial genome sequenced to date at 6.3Mbp. PAO1 is a highly proliferate laboratory strain used in investigations into *Pseudomonas aeruginosa* virulence and biology [31].

Nine strains, NCTC 14642, and NCTC 14644 to NCTC 14651 consecutively, are composed of a mix of *Klebsiella pneumoniae* and *Klebsiella variicola* strains which were all isolated from humans. All carry linear plasmids, a trait which is currently recognised to be an extreme rarity in bacteria; more-so in clinically relevant species [32]. These strains were deposited to facilitate the provision of reference material for the study of linear plasmids and, like many other strains held by the NCTC, will be distributed within the scientific community to elucidate the evolution, significance, prevalence, and relevance of a rare and ostensibly aberrant biological trait.

Of the fifteen strains termed ‘contemporary or significant clinical isolates’, seven (NCTC 14672 – NCTC 14678 consecutively) are isolates of invasive non-typhoidal *Salmonella*, lineages *of Salmonella enterica* subsp. *enterica* endemic in sub-Saharan Africa [33,37]. With the exception of NCTC 14672, all were isolated from instances of bloodstream infection. Invasive non-typhoidal *Salmonella* disease has a high case fatality ratio [38], and it is hoped that the accession and availability of these strains will support an improvement in available diagnostic tools, knowledge of strain pathogenesis, and preventative and curative medicines.

NCTC 14665 *Bordetella pertussis* is the B1917 strain, a member of the ptxP3-ptxA1 (both alleles of the pertussis toxin promoter and pertussis toxin gene respectively) lineage responsible for the resurgence of pertussis in the 1990s, and a contemporaneous model strain for the study of the pathogen [39]. NCTC 14420 to NCTC 14424 consecutively are five reference strains of *Staphylococcus singaporensis* isolated within the same study that described the type strain of the species (NCTC 14419^T^) [5]. NCTC 14401 *Mycoplasma hominis* was isolated from human placenta in 2019. The final member of this group of fifteen clinically associated strains is NCTC 14294 *Yersinia enterocolitica*, one of three strains (the others being NCTC 14295 and NCTC 14298, both *Yersinia enterocolitica*) all from a May 2017 outbreak in the North-West of England. This event was the first detected outbreak of *Yersinia enterocolitica* in England since 10 years prior, and represents the first instance of whole genome sequencing being used to understand and resolve such an outbreak [40].

*Mycoplasma enhydrae* strains NCTC 14347 to NCTC 14350 consecutively [8], *Mycoplasma anatis* strains NCTC 14425 and NCTC 14426, and NCTC 14439 *Mycoplasma miroungigenitalium* are seven veterinary associated strains made available from the NCTC in 2022 [9]. All *Mycoplasma enhydrae* strains were isolated from Southern Sea Otters (*Enhydra lutris nereis*), both *Mycoplasma anatis* strains were isolated from ducks (*Anas platyrhynchos domestica*), and the *Mycoplasma miroungigenitalium* was isolated from a Northern Elephant Seal (*Mirounga angustirostris*). All are reference strains, however the type strains of *Mycoplasma enhydrae* (NCTC 14307^T^) and *Mycoplasma miroungigenitalium* (NCTC 14429^T^) strains were also made available from the NCTC in 2022 as described above.

Twenty-seven strains were accessioned into the NCTC from two different historical/legacy strain archives. Twelve of these strains are *Vibrio cholerae* isolates from an archive held by the UKHSA Gastrointestinal Bacteria Reference Unit (GBRU) and are the first strains from this archive to be listed in the NCTC online catalogue. While accompanying metadata for these strains is scarce, all strains have been serotyped by GBRU; with the exception of NCTC 14081 and NCTC 14085 which are O1 classical and O139 respectively, all strains are non-O1 and non-O139.

The remaining fifteen strains were accessioned from the Murray Collection, a collection of pre-antibiotic era *Enterobacteriaceae* isolates gathered and maintained by Everitt George Dunne Murray (1890 – 1964) [25] and transferred to the NCTC by his son, Robert George Everitt Murray (1919 – 2022). While the geographic origin of all strains is unclear, one strain is known to have been isolated before 1955, one from before 1942, eleven from before 1939, one from before 1936 and one from before 1935. With the exception of NCTC 14117 *Klebsiella pneumoniae*, all are *Salmonella enterica* strains.

## Concluding remarks

With the ongoing support of numerous globally distributed depositors, the portfolio of bacterial strains held by the National Collection of Type Cultures continues to expand and develop. The number and diversity of accessioned strains made available represents significant annual growth for the collection. The accession of the above 125 strains engenders mutualistic benefit for the NCTC, strain depositors, collaborating culture collections, and the global scientific community at large.

The bacterial strains made available from the NCTC in 2022 are exemplary of the collection’s capacity to archive and mobilise useful, emergent, novel, biomedically significant, historically unique, and distinctively rare biological materials. As with all strains held by the NCTC, it is hoped that the above-described strain availability valorises the research of the depositors and furthers the work of other researchers, as well as collaborative and reproducible science more generally.

Ongoing description of medically relevant novel bacterial taxa within the framework of the International Code of Nomenclature of Prokaryotes [41] has continued to increase the number of new type strains the NCTC is able to make available year on year; a trend which the authors of this paper hope to see continue.

More information on depositing bacterial strains into the NCTC, a free service, is available on the NCTC / UKHSA Culture Collections website: https://www.culturecollections.org.uk.

## Abbreviations

AMR: antimicrobial resistance gene
AMRHAI: Antimicrobial Resistance and Healthcare Associated Infections
GBRU: gastrointestinal bacteria reference unit
NCTC: National Collection of Type Cultures
UKHSA: UK Health Security Agency
